# Tibiofemoral dislocation after total knee arthroplasty treated successfully with an external fixation device

**DOI:** 10.1093/jscr/rjad063

**Published:** 2023-02-22

**Authors:** Athanasios Galanis, Eftychios Papagrigorakis, Michail Vavourakis, Panagiotis Karampinas, Christos Vlachos, Christos Patilas, Spiros Pneumaticos, John Vlamis

**Affiliations:** Third Department of Orthopaedic Surgery, National & Kapodistrian University of Athens, KAT General Hospital, Athens, Greece; Third Department of Orthopaedic Surgery, National & Kapodistrian University of Athens, KAT General Hospital, Athens, Greece; Third Department of Orthopaedic Surgery, National & Kapodistrian University of Athens, KAT General Hospital, Athens, Greece; Third Department of Orthopaedic Surgery, National & Kapodistrian University of Athens, KAT General Hospital, Athens, Greece; Third Department of Orthopaedic Surgery, National & Kapodistrian University of Athens, KAT General Hospital, Athens, Greece; Third Department of Orthopaedic Surgery, National & Kapodistrian University of Athens, KAT General Hospital, Athens, Greece; Third Department of Orthopaedic Surgery, National & Kapodistrian University of Athens, KAT General Hospital, Athens, Greece; Third Department of Orthopaedic Surgery, National & Kapodistrian University of Athens, KAT General Hospital, Athens, Greece

## Abstract

Tibiofemoral dislocation after primary total knee arthroplasty (TKA) is a rare but potentially devastating complication with various patient-related and surgeon-related predisposing factors. We present the case of an obese 86-year-old woman who sustained an atraumatic posterior tibiofemoral dislocation 3 days after a primary medial-pivot design TKA. The knee remained unstable after reduction, owing to significant hamstring hypertonia. The administration of botulinum toxin injections in the hamstrings resulted in no clinical improvement. The periprosthetic infection workup was negative and the neurological impairment of the patient was excluded. The patient was reoperated with extensive hamstring release and the application of a lateral external fixator. The external fixator was removed 6 weeks postoperatively, and physical therapy was initiated. At 1-year follow-up, the patient had a painless, stable knee with a 0–100° range of motion, without any neuromuscular impairment.

## INTRODUCTION

Tibiofemoral dislocation after total knee arthroplasty (TKA) is reported in 0.15–0.5% and 3.3% of primary and revision TKAs, respectively [1, 2]. Dislocation can occur both anteriorly and posteriorly, with the posterior ones constituting the vast majority (more than 80% of cases) [2].

The most commonly reported surgeon-related causes include periprosthetic infection, polyethylene wear or fracture, implant malalignment, extensive soft tissue releases during surgery, ligamentous insufficiency, mismatch of the flexion–extension gap, significant preoperative deformities and neuromuscular disorders [3–5].

Predisposing patient-related factors include recent trauma, previous surgeries, connective tissue diseases, inflammatory diseases, diabetes mellitus and obesity.

The diagnosis is usually based on clinical findings. Radiographs may reveal excessive femoral or tibial resection, wrong size or placement of femoral components, and increased tibial slope [6].

## CASE REPORT

**Figure 1:**
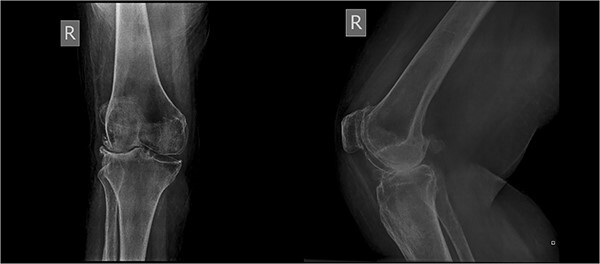
Preoperative X-rays demonstrating stage IV OA.

An 86-year-old female patient with a body mass index (BMI) of 31.2 presented to our outpatient department with severe right knee pain. Physical examination revealed a mechanical block of the knee, limiting its range of motion (ROM) between 10° and 55° of flexion. Plain knee radiographs showed grade 4 knee osteoarthritis (OA) in the Kellgren–Lawrence scale (Fig. 1).

**Figure 2:**
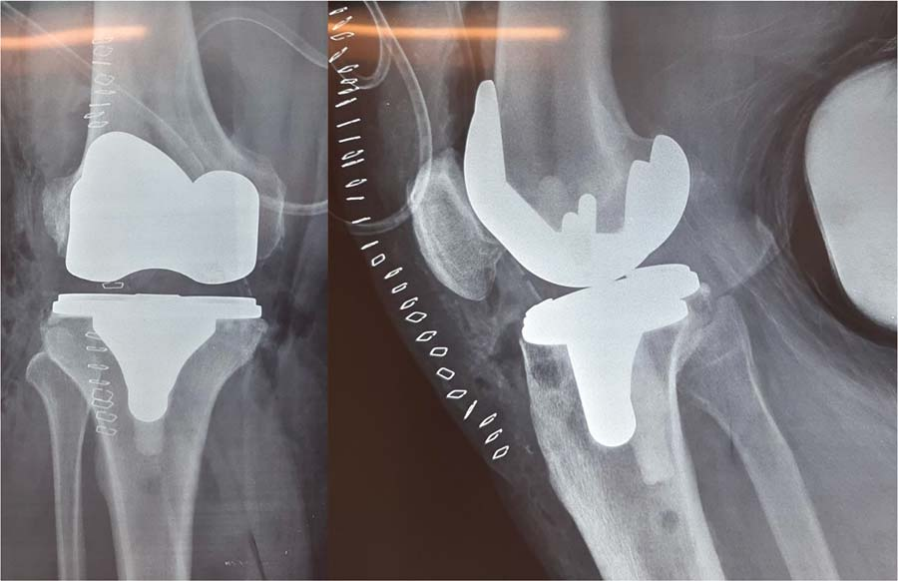
Medial-pivot TKA.

**Figure 3:**
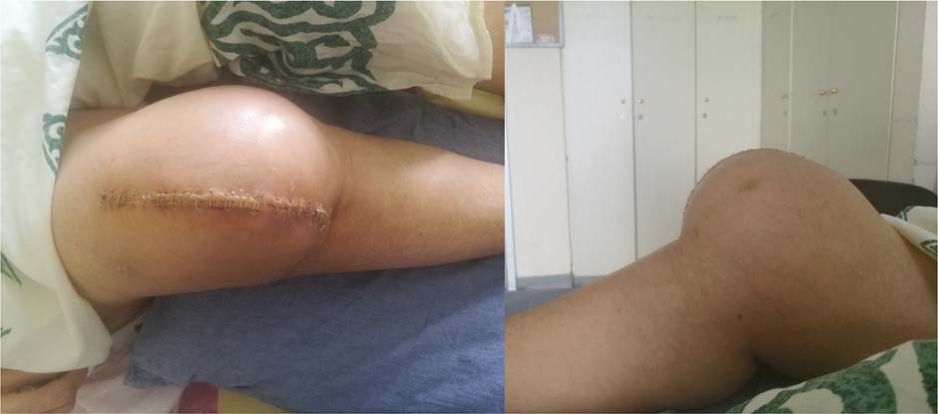
Clinical presentation of tibiofemoral dislocation following TKA.

**Figure 4:**
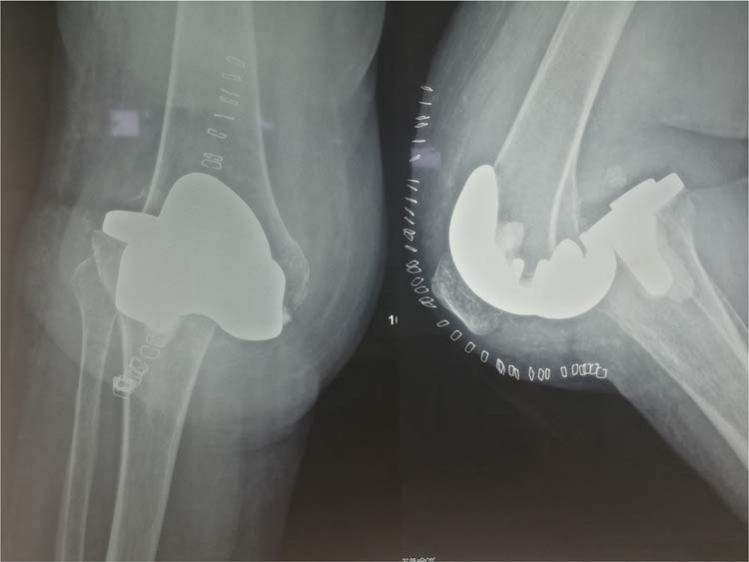
X-rays confirming the dislocation.

The patient underwent a medial-pivot design TKA (Evolution Medial-Pivot Knee System, Microport, China). Intraoperatively, a full ROM was achieved only after extensive soft tissue and bone releases. The extension gap was limited by significant hamstring spasticity, and a proper extension gap was attained after additional anterior femoral cut. Early postoperative recovery was uneventful (Fig. 2). On the third postoperative day, the patient complained of severe knee pain accompanied by excessive swelling during kinesiotherapy. Clinical examination showed a posterior sag sign at 30° of flexion (Fig. 3). No signs of neurovascular deficit were obvious. A knee X-ray revealed posterior TKA dislocation and a small, non-displaced femoral fracture (Fig. 4). The tibial component was reducible, but redislocation occurred with the knee extended over 90°, indicative of severe instability. Laboratory investigations, including erythrocyte sedimentation rate and C-reactive protein, were normal. A knee arthrocentesis was performed with no substantial findings. Two botulinum toxin injections (200 IU) to the hamstrings were performed without improvement. Local muscle hypertonia was evaluated through a neurological examination and a brain computed tomography scan, with no remarkable findings. The patient’s history included intramedullary nailing of the right femur due to an intertrochanteric fracture 8 months before the TKA.

**Figure 5:**
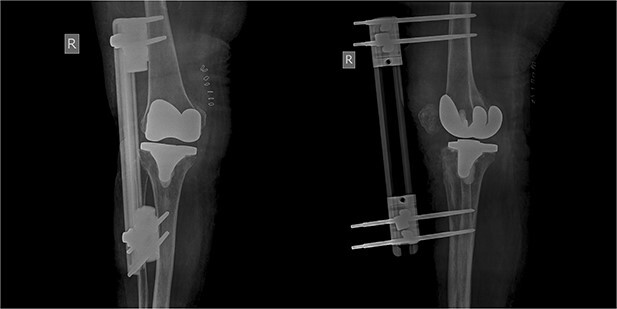
X-rays 1-month after external fixation.

The patient was reoperated, where a more extensive release of the hamstrings was performed, obtaining full extension. The knee was stabilized using a lateral external fixator (Fig. 5). Muscle biopsies were obtained, indicating signs of chronic inflammation. Full weight bearing, using a walker, was permitted from the first postoperative day without substantial complaints.

**Figure 6:**
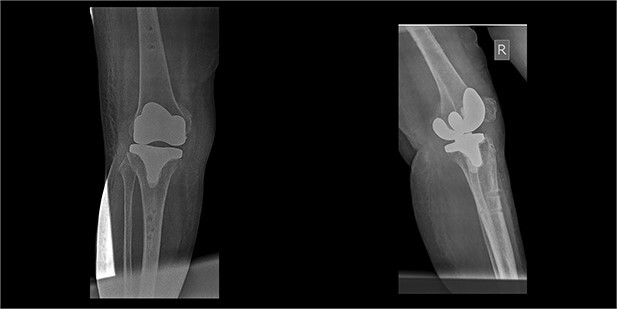
X-rays 3-weeks post removal of external fixator.

**Figure 7:**
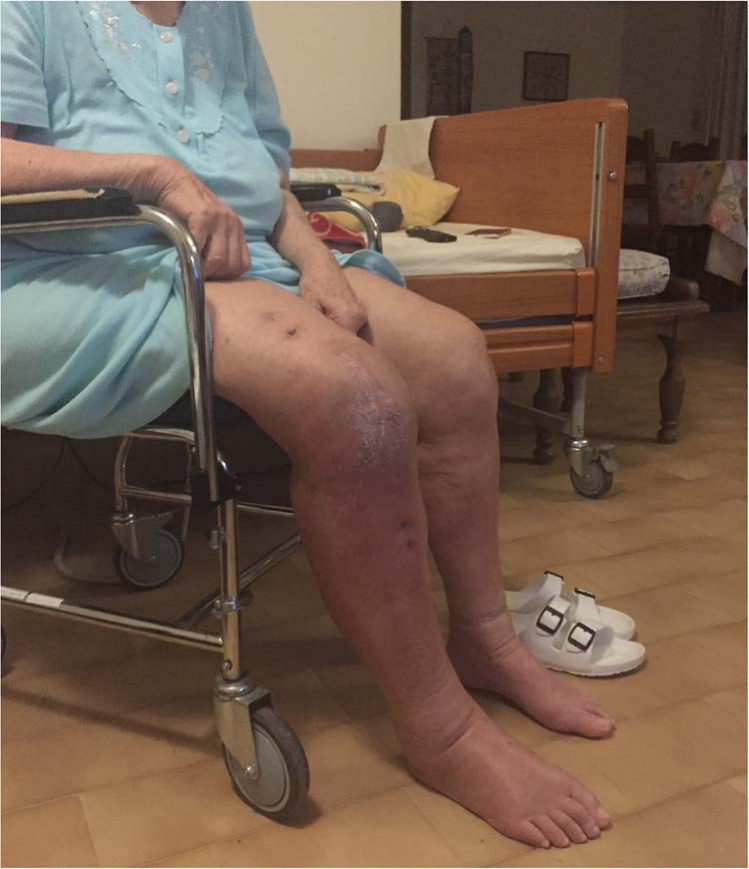
One-year follow-up.

The external fixator was removed after 6 weeks (Fig. 6), and physical therapy was initiated. The patient’s postoperative course was uncomplicated. One week upon removal, 0°–80° of flexion was achieved through everyday kinesiotherapy. On her last visit, 1 year postoperatively, the patient was ambulatory, with knee ROM of 0°–100° (Fig. 7). No further abnormal neuromuscular signs were observed.

## DISCUSSION

The most important risk factors leading to a TKA dislocation include mechanical malalignment, hardware failure, infection, obesity, female gender and muscular imbalance [3, 4]. In our case, there was no apparent mechanical cause, as the prosthesis was well balanced and mechanically aligned intraoperatively. On the contrary, hamstring shortening, which was suspected to be the primary cause of the TKA dislocation, could be attributed to a posterior thigh compartment syndrome secondary to her previous hip surgery [7].

Additionally, some patient-related factors may be implicated. Gender plays an important role, as women are most commonly affected. A recent systematic review reported that 64.4% of patients with TKA dislocation were female [2], while a case series by Jethanandani *et al.* reported an 86% gender relation [4]. One potential reason could be increased ligamentous laxity in females associated with increased estrogen and progesterone levels [8]. Another crucial factor is obesity, which increases the mechanical stress applied to the joints [9]. Obesity is the most frequent comorbidity, with a 40–79% frequency [2, 4]. Furthermore, several case reports have identified obesity as the direct cause of post-TKA dislocation [10]. It correlates with poorer muscle quality and a greater risk of medial collateral ligament avulsion [3, 5]. The mean BMI of patients with tibiofemoral dislocation after TKA has been reported at 33 kg/m^2^ [4]. In our case, the patient was female, obese, with severe preoperative limitation of motion, presenting three patient-related factors that may predispose to dislocation.

Periprosthetic infection is another factor associated with TKA dislocation [4], but the laboratory investigations were negative in our case.

Neuromuscular disorders interfering with agonist–antagonist muscle synergy can be caused by increased muscle tone and spasticity, resulting in knee instability and, eventually, dislocation [11]. Knee instability in these patients may result from increased hamstring spasticity [12]. Our institution’s neurologists meticulously evaluated the patient, but no firm conclusions were reached to explain a potentially catastrophic hamstring muscle spasticity.

In older cruciate-retaining (CR) implants, the reported rate of post-TKA knee instability and dislocation was 1–2% [13]. Nevertheless, no correlation between a medial-pivot TKA and postoperative dislocation was retrieved in the literature.

Definitive management of tibiofemoral dislocation relies on appropriately identifying the potential cause of TKA instability. Conservative treatment through immobilization has been the definitive treatment in 30% of cases, especially when patients are not fit for surgery or in case of early dislocation. Surgical complications are fewer, but the rate of recurrent dislocation is higher. In 70–80% of cases, revision TKA is usually preferred by surgeons. In most cases, both femoral and tibial components are revised. However, there are cases where only the polyethylene was augmented. Revision TKA with increased constraint has proven to be highly successful but bears a high risk of intraoperative and postoperative complications [2, 4].

In the literature, only one case of recurrent TKA anterior dislocation treated with external fixation has been described in an 88-year-old patient with vascular compromise [14]. In our case, using an external fixator proved effective as the definitive treatment, in combination with the appropriate hamstring surgical release. It is a relatively minimally invasive option, suited for a fragile elderly patient, allowing immediate full weight-bearing while protecting a potentially unstable prosthesis.

## CONCLUSION

Tibiofemoral dislocation after primary TKA is a rare but devastating complication, often leading to revision surgery. Orthopedic surgeons should be aware of both patient-related an surgery-related predisposing factors. In our case, external fixation was used for knee immobilization, followed by physical therapy, with satisfactory results at the 1-year follow-up.

## Data Availability

All data are available upon request.
